# Retraction notice to: ‘Monitoring droughts in Eswatini: A spatiotemporal variability analysis using the Standard Precipitation Index’, *Jàmbá: Journal of Disaster Risk Studies* 11(1), a725

**DOI:** 10.4102/jamba.v13i1.1036

**Published:** 2021-03-10

**Authors:** Daniel H. Mlenga, Andries J. Jordaan, Brian Mandebvu

**Affiliations:** 1 Disaster Management Training and Education Centre for Africa, Faculty of Natural and Agricultural Sciences, University of the Free State, Bloemfontein, South Africa; 2 Institute of Development Studies, National University of Science and Technology, Bulawayo, Zimbabwe

**Reason:** The article, Mlenga, D.H., Jordaan, A.J. & Mandebvu, B., 2019, ‘Monitoring droughts in Eswatini: A spatiotemporal variability analysis using the Standard Precipitation Index’, *Jàmbá: Journal of Disaster Risk Studies* 11(1), a725. https://doi.org/10.4102/jamba.v11i1.725, published on 03 October 2019 in *Jàmbá - Journal of Disaster Risk Studies* has been retracted by agreement between the authors (Daniel H. Mlenga, Andries J. Jordaan and Brian Mandebvu), the Publisher (AOSIS) and the journal’s Editor-in-Chief, Prof. Dewald van Niekerk, because of duplicate publication. The second article was published on 24 October 2019 in the *Jàmbá - Journal of Disaster Risk Studies* (https://doi.org/10.4102/jamba.v11i1.712).

We apologise for any inconvenience to the authors and readers of *Jàmbá - Journal of Disaster Risk Studies*.

